# High-resolution monitoring reveals fragmented 24-hour light exposure in intensive care units

**DOI:** 10.1186/s13054-026-06166-8

**Published:** 2026-07-02

**Authors:** Maximilian Niederer, Mathias Bader, Sascha Hammer, Sebastian Labenbacher, Martin Rief, Gabriel Honnef, Philipp Eller, Helmar Bornemann-Cimenti, Paul Zajic

**Affiliations:** 1 Department of Anaesthesiology and Intensive Care, Medical University of Graz, Graz, Austria; 2 Department of Internal Medicine, Medical University of Graz, Graz, Austria

**Keywords:** Intensive care units, Circadian rhythm, Delirium, Critical illness, Sleep wake disorders, Light exposure, Light fragmentation

## Abstract

**Background:**

Light is a key regulator of circadian rhythms, sleep, and neuroendocrine function. Abnormal light exposure has been associated with delirium and adverse outcomes in critically ill patients. While previous ICU studies have generally reported low daytime illuminance and intermittent nocturnal light exposure, most relied on averaged measurements or low temporal resolution monitoring, potentially obscuring clinically relevant exposure patterns. In routine ICU care, light exposure often occurs as brief but frequent events related to necessary interventions, yet the extent and temporal structure of such light fragmentation remain poorly characterized. This study therefore aimed to characterize high-resolution 24-hour light exposure patterns and nocturnal fragmentation in ICU patients.

**Methods:**

In this observational study, ambient illuminance was continuously recorded every 5 s in adult ICU patients over a one-year period. A total of 222 patients were monitored for up to 7 consecutive days, yielding more than 14 million individual measurements. Light exposure was analysed across complete 24-hour cycles and stratified into daytime (07:00–20:59) and nighttime (21:00–06:59) periods. Outcomes included median illuminance, frequency of light events, extreme illuminance (99th percentile), and duration of uninterrupted nocturnal darkness. Mixed-effects models and negative binomial regression were used to account for repeated measurements and to assess associations with room configuration.

**Results:**

Across the 24-hour cycle, median illuminance demonstrated a flattened diurnal pattern, with daytime light levels remaining markedly below those typically encountered in indoor working environments and limited sustained darkness at night. Nighttime exposure was dominated by frequent short-duration light events, resulting in pronounced fragmentation of nocturnal darkness. Fragmentation patterns were largely independent of room configuration and treatment intensity, as ambient illuminance did not differ according to organ support modality once time of day was considered. Extreme illuminance values occurred intermittently, predominantly during daytime, and varied according to room type.

**Conclusions:**

ICU light exposure is characterized primarily by temporal fragmentation rather than sustained brightness. Repeated interruptions of darkness may represent a plausible environmental mechanism contributing to circadian dysregulation. These findings highlight care processes as a key target for interventions and support the use of fragmentation-based metrics in future circadian research in critical care.

**Graphical abstract:**

**Figure:**
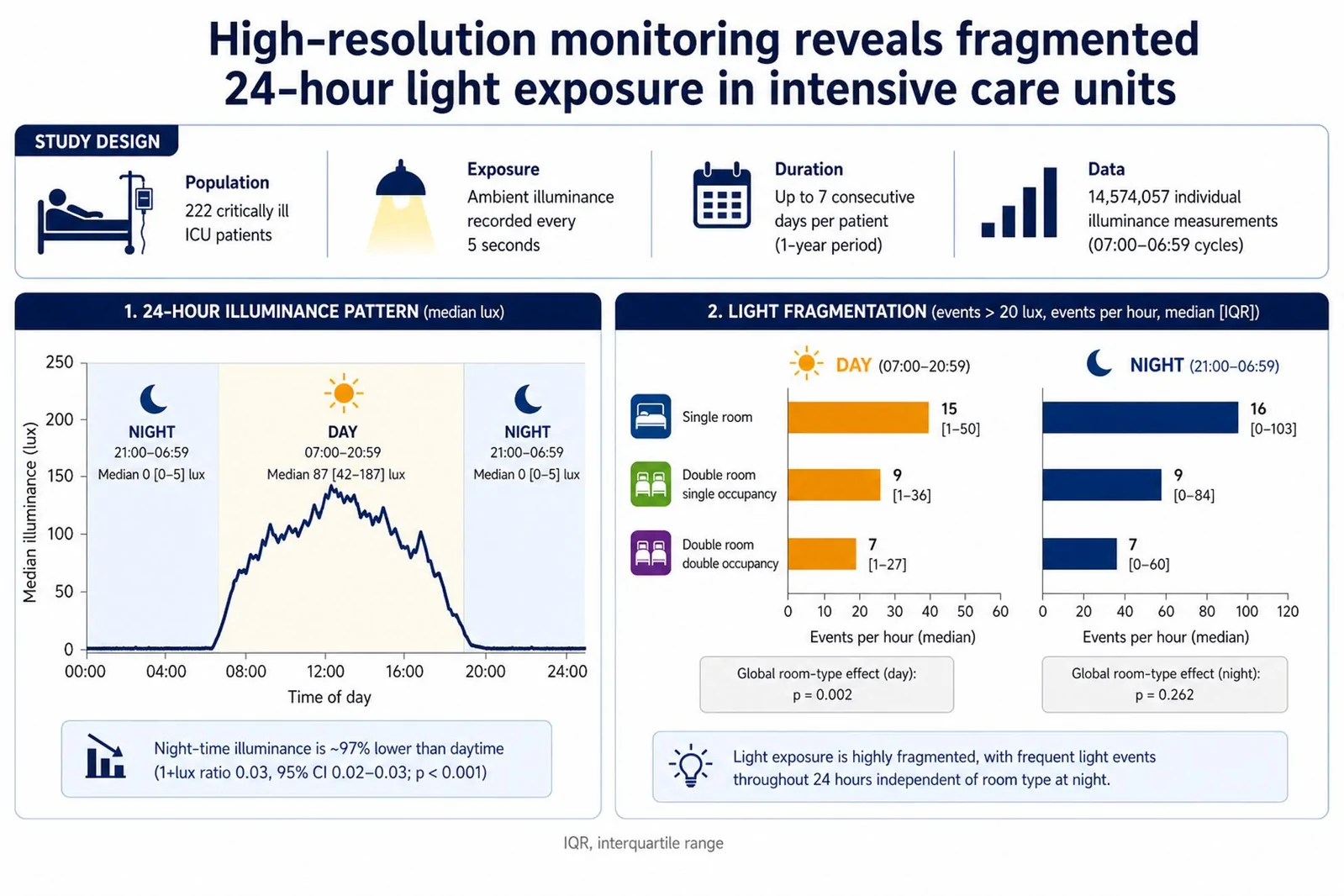

**Supplementary Information:**

The online version contains supplementary material available at https://doi.org/10.1186/s13054-026-06166-8.

## Background

Critically ill patients frequently experience severe sleep disruption and circadian rhythm disturbances, which have been associated with adverse outcomes including delirium, immune dysfunction, metabolic dysregulation, and prolonged recovery [1, 2]. Polysomnographic and observational studies consistently demonstrate that sleep in the intensive care unit (ICU) is highly fragmented, characterized by frequent arousals, reduced slow-wave and rapid eye movement sleep, and a loss of normal circadian sleep-wake organization [1–3].

Environmental factors play a major role in this disruption, with noise, frequent care-related interventions, medications, and abnormal light exposure identified as key contributors [1, 4]. Experimental human studies have shown that mistimed light exposure, including light at night and insufficient daytime illumination, can suppress melatonin secretion, delay or advance circadian phase, and impair sleep continuity, even at relatively low light intensities [5, 6].

Abnormal light exposure has been associated with delirium, immune dysregulation, and delayed recovery in critically ill patients. Observational ICU studies have consistently reported low daytime illuminance and intermittent nocturnal light exposure; however, these investigations were generally limited by small sample sizes and low temporal resolution [7–9]. As a result, most analyses relied on averaged illuminance measures, which may not reflect the clinical reality of ICU care, where light exposure frequently occurs as brief, repeated events related to necessary interventions.

Consequently, a key limitation of existing studies is their reliance on low temporal resolution measurements or averaged illuminance metrics, which obscure the dynamic temporal structure of light exposure. Frequent short-duration light events, even when average illuminance remains low, may repeatedly interrupt darkness and represent a potentially important source of circadian-relevant light exposure. However, the extent of such light fragmentation across complete 24-hour cycles in ICU patients remains poorly characterized.

We therefore conducted a high-resolution observational study using continuous 5-second illuminance measurements over extended ICU stays to characterize 24-hour light exposure patterns, quantify temporal fragmentation of light exposure, and explore how these patterns vary across room configurations. By moving beyond average illuminance metrics, this study aims to provide a more comprehensive and physiologically relevant description of the ICU light environment.

## Methods

### Aim, design and setting

The aim of this study was to characterize the 24-hour light environment in ICUs, with particular focus on nocturnal light fragmentation and extreme illuminance events, and to assess the influence of room occupancy on these patterns.

We conducted an observational cohort study across three adult ICUs at a tertiary academic hospital in Graz, Austria. The study was non-interventional, and all measurements were performed during routine clinical care without modification of patient management or ward workflows. Two of the participating ICUs were commissioned in 2017 and the third in 2024. All units shared an identical architectural layout, consisting of a central nursing station surrounded by patient rooms arranged in a U-shaped configuration.

Patient rooms featured large windows with varying orientations (east, south, or west), allowing substantial natural daylight exposure. Beds were not directly oriented toward the windows, but patients were exposed to ambient daylight within the room. Monitoring equipment was typically positioned beside the bed. Artificial lighting consisted of dimmable ceiling lights that could be adjusted according to clinical needs, resulting in a mixed natural and artificial light environment.

Potential sources of bias were minimized through automated continuous recording and predefined analytical procedures.

### Participants and data sources

Consecutively admitted adult ICU patients were eligible for inclusion if valid light measurement data were available. Patients without valid or complete light sensor recordings, including those with device malfunction or insufficient recording duration, were excluded from the analysis (Supplementary Figure S1).

Ambient light exposure was recorded using calibrated illuminance sensors (SDL400 Light Meter, Extech Instruments Corp., Nashua, NH, USA). The devices were positioned at patient eye level and recorded illuminance every 5 s, yielding more than 14 million individual observations. Illuminance values were stored in lux (lx).

Room occupancy information was extracted from clinical metadata and classified per patient-day as single room, double room with single occupancy, or double room with double occupancy. Room occupancy was treated as a time-varying exposure and linked to light measurements on a per-patient, per-day basis.

### Data processing and definitions

All timestamps were standardized to local time (Europe/Vienna). A 24-hour day was defined from 00:00 to 23:59. For the primary analysis, daytime was defined as 07:00–20:59 and nighttime as 21:00–06:59, reflecting routine clinical workflows and ward lighting schedules in the participating ICUs.

For each patient-day, light exposure metrics were calculated separately for day and night periods, including median illuminance, mean illuminance, 95th and 99th percentiles, cumulative minutes above 20 lx, and number of light events. Light events were defined as transitions from ≤ 20 lx to > 20 lx. The threshold of 20 lx was selected a priori as a pragmatic indicator of meaningful nocturnal light exposure based on previous circadian and sleep research demonstrating that relatively low indoor light intensities can influence circadian physiology, particularly when exposures are repeated or occur during the biological night. In addition, illuminance thresholds in the range of 10–30 lx have been used in prior investigations of hospital and ICU light environments to distinguish background darkness from care-related light exposure [6, 10].

To assess the robustness of the selected threshold, sensitivity analyses were performed using alternative thresholds of 10 lx and 50 lx. These analyses were prespecified to evaluate whether observed fragmentation patterns were dependent on the chosen event definition.

No minimum duration criterion was applied. This approach was intentionally chosen to maximize sensitivity for detecting interruptions of darkness and to provide a descriptive characterization of temporal light fragmentation. We acknowledge that some detected events may represent brief increases in illuminance of uncertain physiological relevance.

Although biologically weighted metrics such as melanopic equivalent daylight illuminance may provide a more physiologically relevant representation of circadian light exposure, spectral information was not available from the recording devices used in this observational study. Consequently, conventional photopic illuminance (lux) represented the only continuously available exposure measure. Likewise, duration-weighted or biologically weighted event definitions may offer additional insights but currently lack standardized thresholds for ICU research.

### Statistical analysis

Descriptive statistics are presented as medians with interquartile ranges (IQRs) unless otherwise specified.

To account for repeated measurements within individuals, mixed-effects models were used throughout, with patient ID included as a random intercept. Continuous outcomes were analyzed using linear mixed-effects models with log-transformed illuminance values where appropriate. Count outcomes were analyzed using generalized linear mixed-effects models with a negative binomial distribution due to overdispersion.

Interaction effects between room occupancy and period (day vs. night) were formally tested. Post-hoc pairwise comparisons were performed using estimated marginal means with Holm correction for multiple testing. Effect sizes for count models are reported as incidence rate ratios with 95% confidence intervals.

Given the observational design and exhaustive inclusion of available measurements, no formal a priori power calculation was performed.

All analyses were conducted in R (version 4.5.2, R Foundation for Statistical Computing, Vienna, Austria) using the packages lme4, glmmTMB, emmeans, and tidyverse. Statistical significance was defined as a two-sided p value < 0.05.

Missing data were minimal due to continuous automated recording and were not expected to materially influence the results; consequently, no formal imputation was performed.

## Results

In this high-resolution observational study 14,574,057 individual illuminance measurements were sampled. Across 222 critically ill patients, ambient light exposure in the ICU followed a pronounced diurnal pattern.

**Table 1:** Baseline patient characteristics, admission details, and light-exposure observation metrics

Characteristics	Overall
Age, median (IQR)	67 (58–75)
Male sex, n (%)	132 (59.5)
BMI, median (IQR), kg/m^2^	26.8 (23.9–30.5)
Admission reason	
Medical	49 (22.1)
Surgical	113 (50.9)
Trauma	53 (23.9)
Neurological	6 (2.7)
Other	1 (0.4)
ICU days observed per patient, median (IQR)	5 (4–8)
Total observation days, n	2,158
Day periods, n	1,069
Night periods, n	1,089
SAPS III, median (IQR)	50.4 [25.7–69.1]
Total illuminance measurements, n	14,574,057
Sampling interval, s	5

**Fig. 1:**
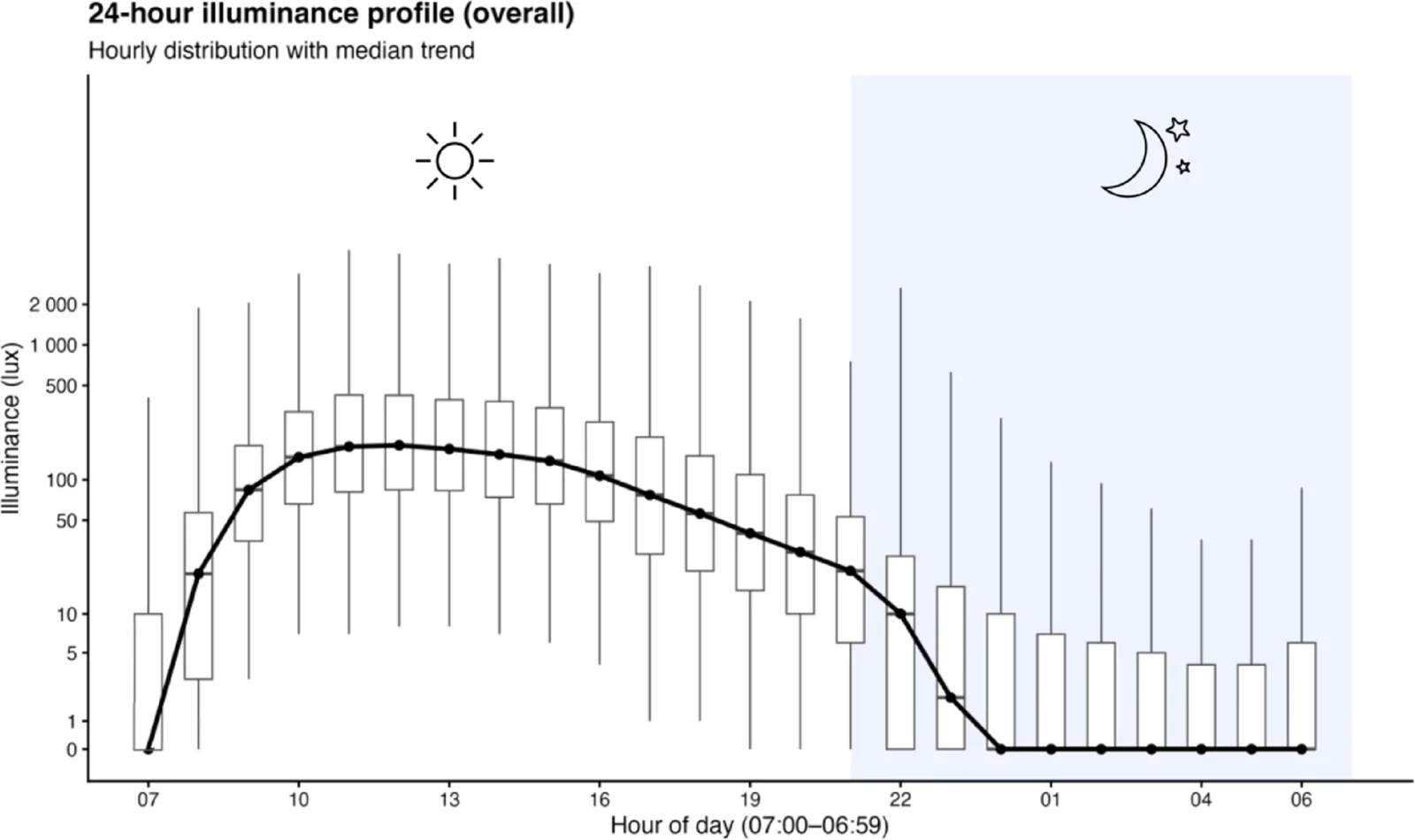
Twenty-four-hour illuminance profile across the ICU stay. Distribution of ambient illuminance aggregated across all patients. Boxplots represent hourly distributions, and the solid black line indicates the hourly median trend. The time axis is shifted to start at 07:00 and end at 06:59 to preserve a single day-night transition. Night-time periods (21:00–06:59) are shaded in blue. Illuminance is displayed on a logarithmic scale

Baseline patient characteristics, admission details, and observation metrics are summarised in Table 1. Median illuminance at the patient-period level was 86.5 [42.0-187.0] lux during daytime hours (07:00–20:59; 1,069 patient-days) and decreased to 0 [0–5] lux during night-time (21:00–06:59; 1,089 patient-nights). These daytime illuminance levels were substantially lower than those commonly reported for office environments (approximately 300–500 lx) and remained far below typical outdoor daylight exposure. The highest hourly median illuminance was observed around

midday, peaking at approximately 12:00 (180 lx). In a mixed-effects model with patient-level random intercepts and log-transformed median illuminance as the outcome, night-time illuminance was markedly lower than daytime illuminance, corresponding to a (1 + lux) ratio of 0.03 (95% CI 0.02–0.03; *p* < 0.001), indicating minimal sustained ambient light exposure during most nocturnal periods (Fig. 1).

Despite low median night-time illuminance, light exposure above 20 lx occurred frequently throughout the 24-hour cycle. During daytime, median rates of discrete light events exceeding 20 lx were 15 [1–50] events per hour in single rooms, 9 [1–36] events per hour in double rooms with single occupancy, and 7 [1–27] events per hour in double rooms with double occupancy. Event rates differed significantly across room configurations during daytime (global room-type effect *p* = 0.002). Comparable event rates were observed during night-time, with medians of 16 [0-103], 9.00 [0–84], and 7 [0–60] events per hour, respectively, without a significant overall difference between room types (global room-type effect *p* = 0.262).

**Fig. 2:**
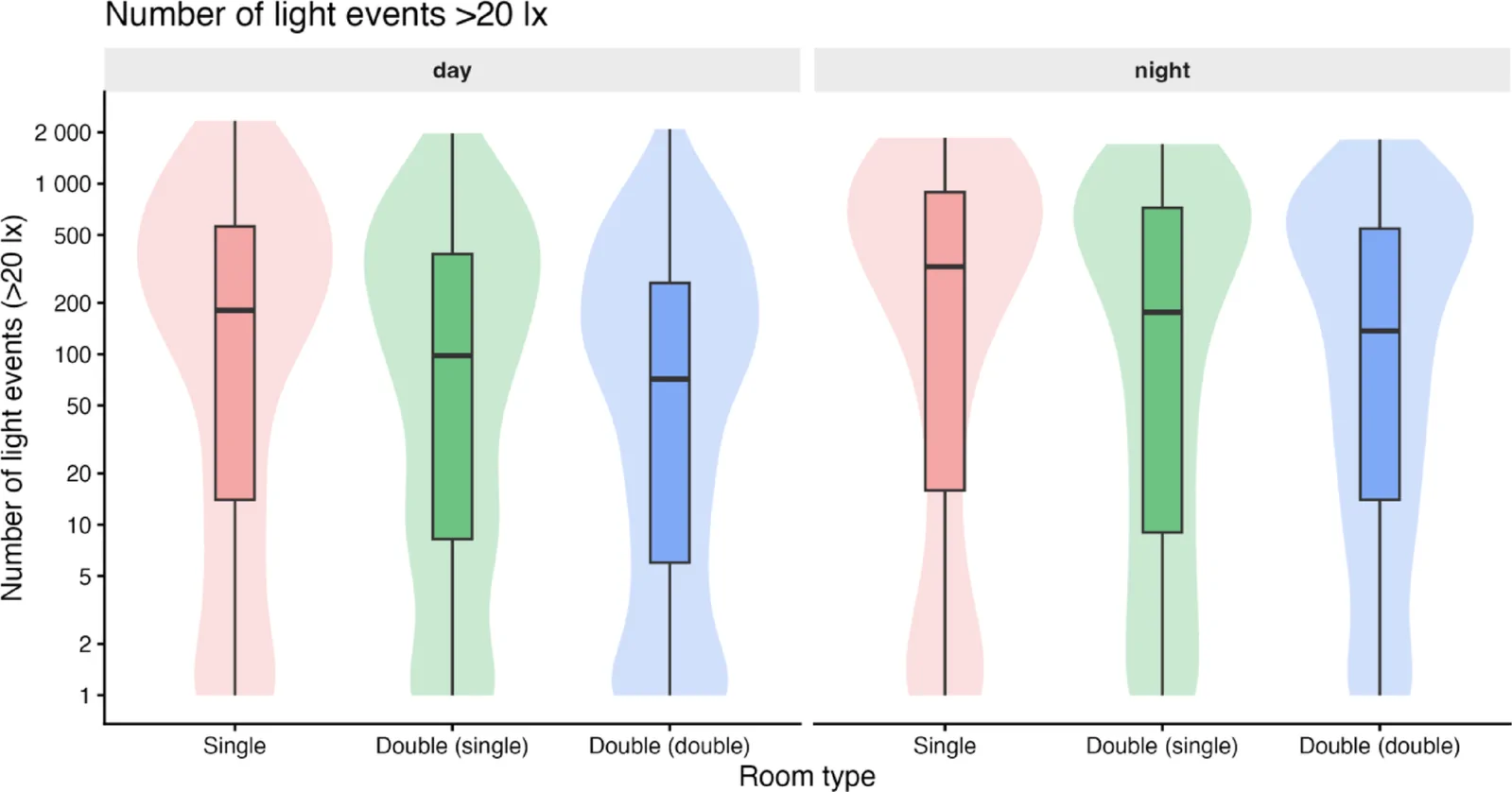
Distribution of light fragmentation events exceeding 20 lx, stratified by room type and time of day. Violin plots with embedded boxplots illustrate the number of discrete light events per patient-day (daytime) and per patient-night (night-time)

Detailed temporal patterns across ICU stay are shown in the Supplementary Material (Supplementary Figure S2) (Fig. 2).

**Fig. 3:**
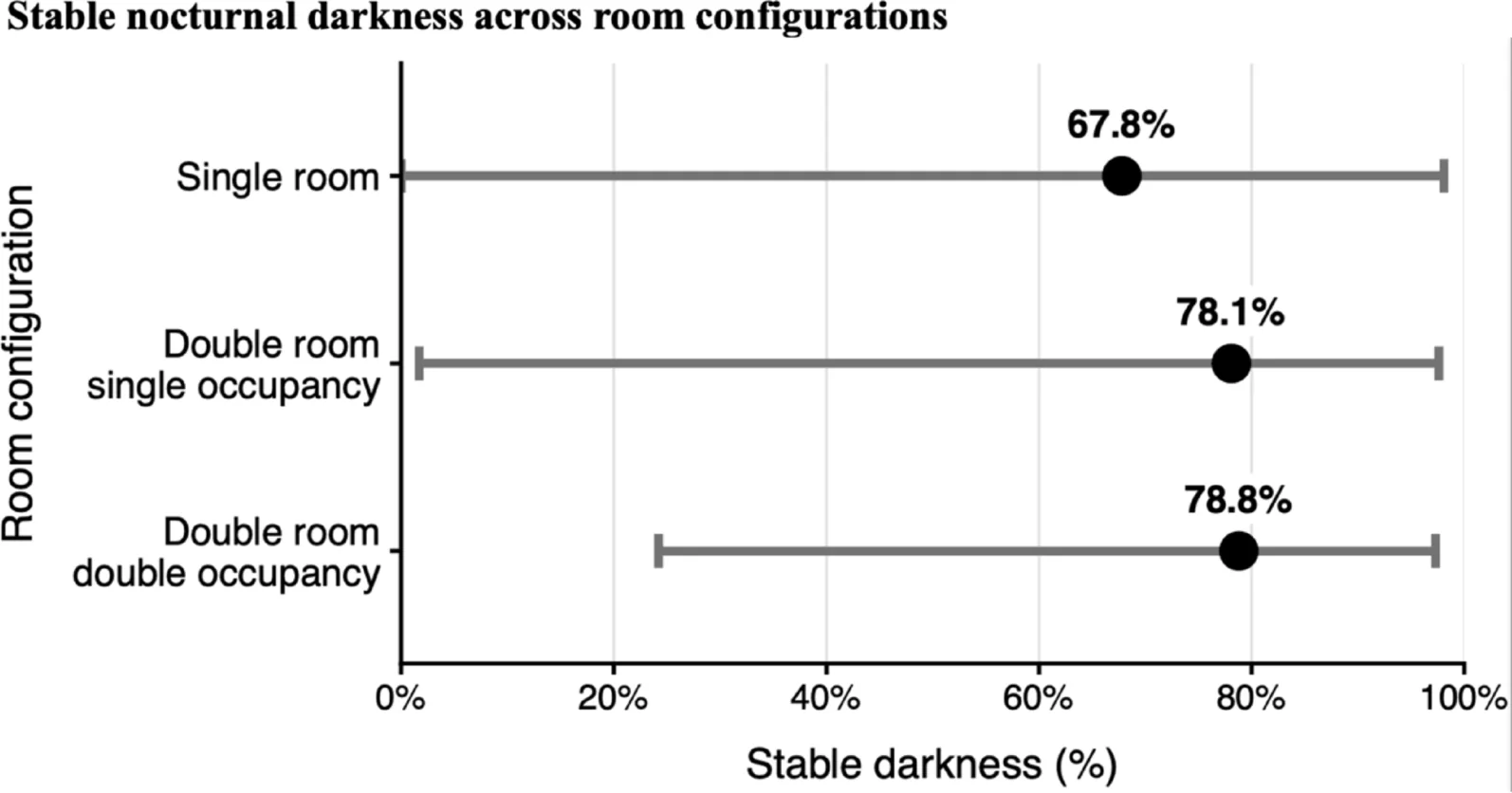
Points indicate median percentages of nighttime spent in uninterrupted darkness, defined as illuminance ≤20 lx for at least 5 consecutive minutes. Horizontal whiskers represent interquartile ranges. No statistically significant association between room configurations and stable nocturnal darkness was observed in the beta mixed-effects model (global p = 0.166)

The amount of stable nocturnal darkness varied modestly according to room configuration. The proportion of night-time spent in uninterrupted darkness at illuminance levels ≤ 20 lx for at least 5 min was 67.8 [0.0-98.1] % per patient-night in single rooms, compared with 78.1 [1.7–97.6] % in double rooms with single occupancy and 78.8 [24.2–97.3] % in double rooms with double occupancy (Fig. 3).

A ≥ 5-minute threshold was chosen to capture clinically meaningful periods of sustained darkness likely relevant for circadian regulation and sleep continuity, as very brief light interruptions are common in ICU care and may have limited physiological impact. This pragmatic cut-off aligns with circadian research emphasizing the importance of duration and continuity of darkness rather than brief fluctuations in light exposure [10]. However, these differences did not reach statistical significance in a beta mixed-effects model with logit link and patient-level random intercepts (global room-type effect *p* = 0.166).

Ambient light exposure was not influenced by the intensity of organ support after accounting for time of day. In mixed-effects models including invasive mechanical ventilation, non-invasive ventilation, and renal replacement therapy as time-varying covariates, none of these factors showed meaningful associations with ambient illuminance. Estimated effects were small and statistically non-significant for invasive ventilation (β = 0.045, SE 0.056), non-invasive ventilation (β = −0.040, SE 0.084), and renal replacement therapy (β = −0.063, SE 0.101). Descriptive analyses confirmed largely overlapping illuminance distributions across support categories during both day and night. These findings indicate that the ICU light environment was primarily determined by temporal factors rather than treatment intensity or patient acuity.

**Fig. 4:**
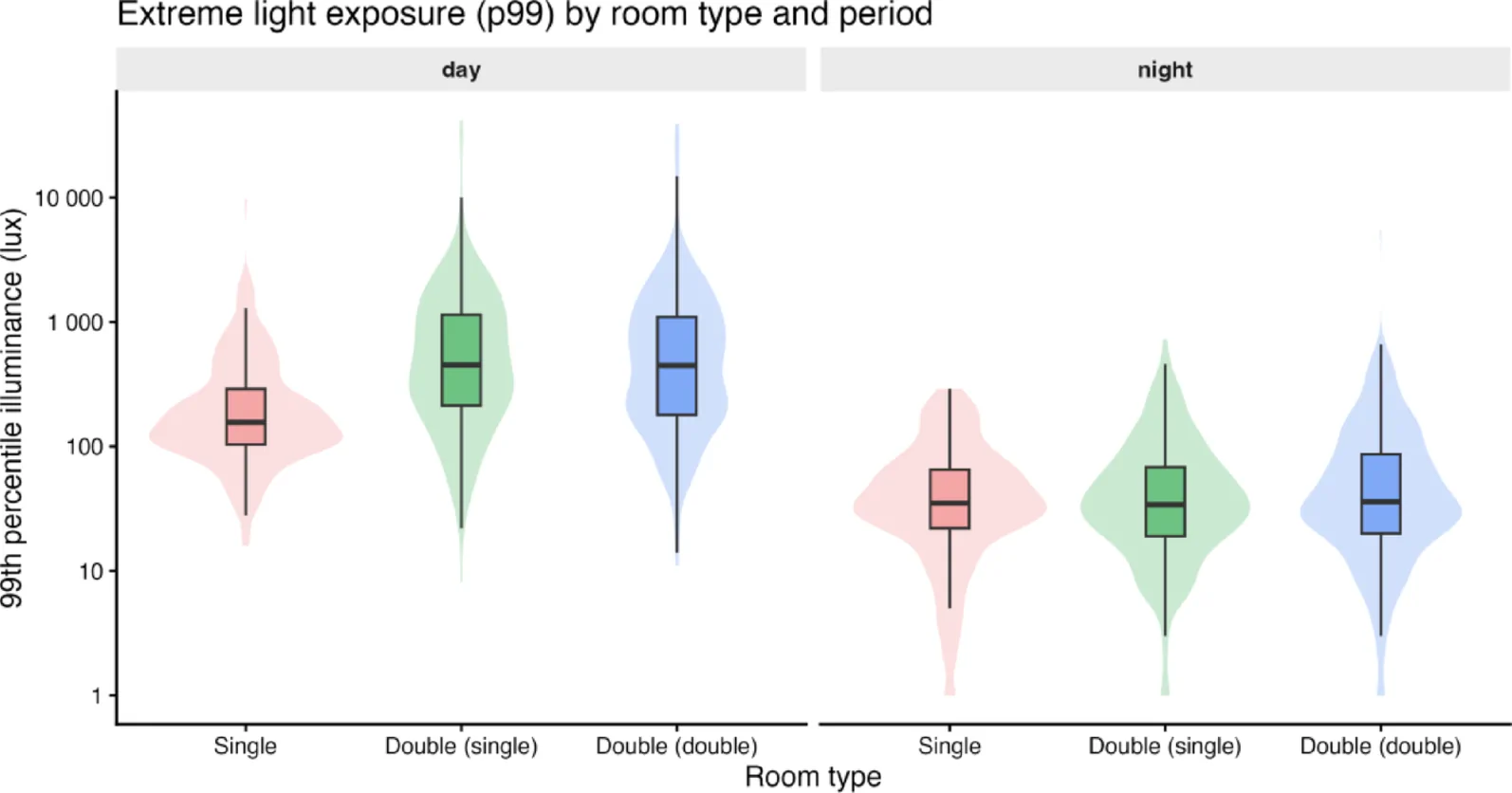
Extreme light exposure by room type and period. 99th percentile (p99) of illuminance was calculated per patient-day and per patient-night to characterize extreme light exposure. Violin plots with embedded boxplots display the distribution of p99 values

Sensitivity analyses using alternative thresholds are shown in the Supplementary Material (Supplementary Figure S3). Fragmentation patterns remained qualitatively consistent across all investigated thresholds, indicating that the observed temporal structure of nocturnal light exposure was not driven by the specific threshold selected for event definition (Fig. 4).

Extreme light exposure, quantified as the 99th percentile illuminance (p99) per patient-period, differed substantially across room configurations during daytime. Median p99 values were 156 [103–284] lux in single rooms, 450 [213–1141] lux in double rooms with single occupancy, and 446 [179–1097] lux in double rooms with double occupancy (global effect *p* < 0.001). Given the comparable findings between double rooms irrespective of occupancy, these differences are unlikely to reflect patient number per se but rather structural and environmental factors such as window exposure, spatial layout, and positioning of light sources. At night, extreme illuminance levels were markedly lower and more homogeneous across room types (p99 ~ 34–35 lx; global effect *p* = 0.072), supporting the notion that daytime extremes are primarily driven by ambient environmental conditions, whereas nocturnal light exposure is dominated by care-related light events independent of room configuration. Corresponding graphical representations of these findings are provided in the Supplementary Material.

Seasonal variation in ambient light exposure is presented in the Supplementary Material (Supplementary Figure S4).

## Discussion

In this high-resolution observational study comprising more than 14 million illuminance measurements, we demonstrate that light exposure in the ICU is defined primarily by temporal fragmentation across the 24-hour cycle rather than by sustained brightness.

Despite low median nocturnal illuminance and an overall diurnal pattern, nighttime conditions were repeatedly disrupted by short-duration light events, resulting in limited periods of uninterrupted darkness. These dynamic exposure characteristics are not captured by conventional summary metrics and represent a fundamental feature of the ICU light environment. Importantly, the convergence of fragmentation patterns across room types, treatment intensities, and temporal analyses suggests that nocturnal light exposure in the ICU represents a structural characteristic of care delivery rather than an incidental environmental factor.

The robustness of these findings is supported by multiple complementary analyses enabled by the high temporal resolution of the dataset. Fragmentation patterns remained consistent across the full distribution of illuminance values, across alternative threshold definitions, and across different representations of temporal structure, including bout duration and cumulative exposure metrics. This consistency suggests that nocturnal light fragmentation represents a fundamental characteristic of ICU light environments rather than an artefact of a single analytical approach.

Circadian physiology depends not only on absolute light intensity but also on the temporal stability of light-dark cycles. Experimental studies have demonstrated that even brief light exposures during the biological night can suppress melatonin secretion and induce circadian phase shifts, particularly when exposures are repeated or occur at sensitive circadian phases [11, 12]. The fragmented nocturnal light patterns observed in this cohort may represent a plausible environmental mechanism contributing to circadian disruption in critically ill patients, even in the absence of high average light levels. This interpretation is consistent with the pronounced sleep fragmentation and circadian misalignment that have been repeatedly documented in ICU populations [1, 3, 13]. Recent reviews have highlighted sleep and circadian disruption as pervasive features of critical illness and identified environmental modification as a promising target for future ICU interventions [14].

Previous investigations of ICU light exposure have largely relied on mean or median illuminance values. While these studies consistently describe low daytime light levels and intermittent nocturnal illumination, associations with circadian or sleep-related outcomes have remained inconsistent [6, 7]. Our findings extend this literature by demonstrating that nocturnal light exposure accrues predominantly through frequent short interruptions of darkness rather than sustained illumination. As a result, static intensity-based metrics substantially underestimate potentially circadian-relevant exposure in the ICU.

An additional finding deserving consideration is the remarkably low daytime illuminance observed throughout the cohort. Median daytime illuminance was only 86.5 lx, with peak hourly median values remaining below levels commonly encountered in office environments and far below typical outdoor daylight exposure. From a circadian perspective, insufficient daytime light exposure may reduce the strength of environmental entrainment signals and thereby contribute to circadian dysregulation independently of nocturnal light exposure. This interpretation is supported by recent observations demonstrating persistently low daytime illuminance levels in modern ICUs, suggesting that inadequate daytime light exposure may remain an underrecognized barrier to circadian entrainment despite advances in ICU design [9]. Accordingly, the ICU light environment observed in this study may be characterized by a dual challenge: limited daytime light availability combined with frequent interruptions of nocturnal darkness. Future interventional studies should evaluate whether optimizing both daytime light exposure and nighttime darkness provides greater circadian benefit than targeting either component alone.

Light events in this study were defined using a threshold-based approach (> 20 lx) without a minimum duration requirement, allowing sensitive detection of transient increases in illuminance. Although such an approach may increase event counts in high-frequency data and may capture brief illuminance fluctuations of uncertain physiological relevance, it enabled sensitive detection of interruptions of darkness across the entire observation period. Importantly, the overall interpretation was supported by complementary metrics, including cumulative exposure time, stable darkness duration, and extreme illuminance measures, all of which demonstrated consistent evidence of nocturnal light fragmentation. Future work may refine event definitions by incorporating duration thresholds or biologically weighted measures, but the present data demonstrate that frequent short light interruptions are a dominant characteristic of the nocturnal ICU environment, consistent with experimental evidence on circadian light sensitivity [12, 15, 16].

Room configuration influenced daytime illuminance and extreme light exposure but had only limited effects on nocturnal fragmentation and sustained darkness. Similar fragmentation patterns across room configurations indicate that architectural layout alone is unlikely to explain the observed nocturnal light exposure patterns. Nevertheless, environmental design remains clinically relevant. Recent work has demonstrated that ICU room modifications incorporating circadian-oriented lighting concepts may influence melatonin profiles and delirium outcomes, highlighting the potential interaction between architectural design and circadian health [17]. The persistence of nocturnal fragmentation across room configurations suggests that nighttime light exposure is driven predominantly by care-related activities and workflow patterns rather than architectural layout alone. Single rooms are often allocated to patients requiring closer monitoring, isolation, or advanced organ support, which has been associated with increased frequency of nighttime interventions in prior ICU studies [1, 4]. Consistent with this practice, patients allocated to single rooms in our cohort did not differ significantly between room types (Supplementary Table S1). In contrast, the use of invasive ventilation and renal replacement therapy varied across room configurations, whereas non-invasive ventilation rates were low in all groups. Despite these differences, nocturnal fragmentation patterns remained broadly similar across room configurations.

Importantly, additional analyses demonstrated that the intensity of organ support itself did not influence ambient light exposure once time of day was considered. Neither invasive ventilation, non-invasive ventilation, nor renal replacement therapy showed meaningful associations with illuminance levels. These analyses by organ support modalities are provided in the Supplementary Material (Supplementary Figures S5–S7). Likewise, nocturnal light fragmentation did not differ significantly across sedation categories (Supplementary Figure S8), further supporting the interpretation that the observed fragmentation primarily reflects structural characteristics of ICU care processes rather than patient-specific treatment intensity or sedation depth. This finding strengthens the interpretation that nocturnal light exposure in the ICU is primarily determined by system-level workflow patterns rather than patient-specific treatment complexity. Consequently, strategies aiming to improve circadian conditions may need to focus on unit-wide care processes rather than targeting selected patient groups.

Extreme illuminance values, reflected by elevated 99th percentile levels, occurred intermittently and were most prominent during daytime. These events are plausibly attributable to intervention-related light sources, such as examination lamps or procedural lighting. While such exposures are clinically relevant, they were infrequent and do not explain the pervasive nocturnal fragmentation observed across the cohort. Accordingly, extreme illuminance metrics should be interpreted as secondary descriptors of the ICU light environment rather than primary drivers of circadian disruption, in line with previous ICU lighting studies [6–8].

These findings have important implications for both research and clinical practice. First, studies assessing ICU light exposure should incorporate fragmentation-based metrics to more accurately capture biologically relevant exposure patterns. Second, interventions aiming to improve circadian conditions should not focus exclusively on architectural redesign or increasing daytime illumination. The present findings suggest that reducing the frequency of nocturnal light interruptions may represent a feasible and immediately actionable target within existing ICU infrastructures.

Emerging evidence suggests that multicomponent non-pharmacological interventions targeting environmental factors and circadian regulation may improve sleep quality and reduce delirium risk in critically ill patients [18]. Potential strategies include clustering nighttime care activities, minimizing unnecessary room entries, reducing monitor and workstation light spill, using task-directed lighting during procedures, and implementing structured “dark periods” whenever clinically appropriate. Such interventions may be easier to implement than large-scale environmental modifications and could substantially improve preservation of nocturnal darkness. Future studies should evaluate whether these workflow-based approaches translate into measurable improvements in circadian physiology, sleep quality, delirium, and recovery.

Prior interventional studies have primarily emphasized daytime light augmentation, with variable effects on sleep and delirium [8, 19]. Our results suggest that preserving uninterrupted nocturnal darkness may represent an important target for future interventions aiming to improve circadian conditions in critically ill patients.

In conclusion, the ICU light environment is characterized by pronounced temporal fragmentation across the entire 24-hour cycle. Traditional average illuminance measures fail to capture frequent short interruptions of nocturnal darkness and therefore substantially underestimate circadian-relevant exposure. By shifting the focus from static intensity metrics to dynamic exposure characteristics, this study provides a more physiologically meaningful framework for understanding and ultimately improving the ICU light environment.

### Strengths

This study has several notable strengths. First, it represents one of the largest and highest-resolution investigations of light exposure in the ICUs to date, comprising more than 14 million individual illuminance measurements collected at 5-second intervals across over 2,000 day-night periods. This dense temporal sampling allowed detailed characterization of both average light exposure and dynamic exposure patterns, including light-event frequency, cumulative exposure time, and uninterrupted darkness.

Second, the longitudinal design with repeated measurements across multiple consecutive ICU days per patient enabled robust within-patient comparisons while accounting for interindividual variability. The use of mixed-effects models appropriately addressed clustering at the patient level and strengthened the robustness of the observed associations and characterization of environmental exposure patterns.

Third, the study goes beyond traditional summary metrics by introducing fragmentation-based measures of nocturnal light exposure, such as the frequency of light events and the duration of uninterrupted darkness. These metrics capture physiologically relevant exposure characteristics that are not reflected by mean or median illuminance alone and align with established concepts of sleep and circadian disruption.

Fourth, room allocation was incorporated on a day-by-day basis, allowing differentiation between single rooms, double rooms with single occupancy, and double rooms with double occupancy. This granularity enabled evaluation of architectural and organizational influences on light exposure across the full 24-hour cycle.

### Limitations

Several limitations should be acknowledged. First, this was a single-center observational study, which may limit generalizability to ICUs with different architectural layouts, lighting systems, or care routines. However, the observed exposure patterns are consistent with reports from other ICUs, supporting external validity.

Second, illuminance was measured as ambient light intensity (lux) and did not capture spectral composition or melanopic irradiance, which are more directly linked to circadian phototransduction. As a result, biological effects on circadian phase or melatonin secretion can only be inferred indirectly.

Third, although extreme illuminance values were intentionally retained to reflect real-world care activities, such as procedures requiring high-intensity lighting, the study did not differentiate between task-related and incidental light exposure, nor did it systematically document the clinical context of light events.

Fourth, neither biomarkers of circadian function (e.g., melatonin secretion or core body temperature) nor patient-centered outcomes such as sleep quality, delirium, or recovery trajectories were assessed. Therefore, the present study characterizes environmental light exposure patterns rather than their direct physiological or clinical consequences. Future studies integrating high-resolution light monitoring with objective assessments of circadian function, sleep, and patient outcomes are needed to clarify the biological relevance and potential clinical impact of nocturnal light fragmentation.

The definition of light events was based on threshold crossings without a minimum duration requirement. Consequently, some detected events may have reflected transient illuminance fluctuations, such as reflections, monitor-related light changes, or brief care-related exposures, whose physiological significance remains uncertain. Although complementary analyses supported the overall findings, future studies should evaluate duration-weighted and biologically informed event definitions to better distinguish circadian-relevant light exposure from transient environmental fluctuations.

Room allocation was determined according to routine clinical practice rather than study procedures. Consequently, residual confounding related to illness severity, monitoring requirements, or isolation precautions cannot be excluded when interpreting comparisons between room configurations.

## Conclusions

In this high-resolution cohort, the light environment was characterized primarily by temporal fragmentation with repeated interruptions of darkness, largely independent of room configuration or treatment intensity. Light exposure in the ICU appears to be a structural byproduct of care processes rather than a purely environmental variable. These findings suggest that workflow-driven light patterns may represent an important environmental factor potentially contributing to circadian disruption in critically ill patients. Consequently, efforts to improve the circadian environment should extend beyond architectural design and daylight exposure alone and also consider workflow-based measures aimed at preserving nocturnal darkness, such as clustering nighttime interventions and minimizing unnecessary light exposure. Future interventions should address both the preservation of nocturnal darkness and the provision of adequate daytime light exposure to support circadian entrainment in critically ill patients.

## Electronic Supplementary Material

Below is the link to the electronic supplementary material.

Additional file 1.

Additional file 2.

## Data Availability

The datasets used and/or analysed during the current study are available from the corresponding author on reasonable request.

## Abbreviations

ICU: Intensive care unit
Lx: Lux
p99: 99th percentile of illuminance
IQR: Interquartile range
IRR: Incidence rate ratio
CI: Confidence interval
NB: Negative binomial (regression)
GLMM: Generalized linear mixed-effects model
LMM: Linear mixed-effects model
SD: Standard deviation
RR: Relative risk
RCT: Randomized controlled trial
IMV: Invasive mechanical ventilation
NIV: Non-invasive ventilation
