# Supplementary material for: High-resolution monitoring reveals fragmented 24-hour light exposure in intensive care units
Source: Crit Care. 2026 Jul 2;30:485. doi: 10.1186/s13054-026-06166-8 (PMC13595780; doi:10.1186/s13054-026-06166-8)
Supplement: Supplementary file 1 [file 13054_2026_6166_MOESM1_ESM.docx]

# STROBE checklist for reporting of observational studies

| Section | Item | Recommendation | Reported on page |
| --- | --- | --- | --- |
| Title and abstract | 1a | Indicate study design in title or abstract | Title; Abstract |
| Title and abstract | 1b | Provide structured abstract with key elements | Abstract |
| Introduction | 2 | Explain scientific background and rationale | Background |
| Introduction | 3 | State specific objectives | End of Background |
| Methods | 4 | Present key elements of study design | Aim, design and setting |
| Methods | 5 | Describe setting, locations, and dates | Aim, design and setting |
| Methods | 6a | Give eligibility criteria and selection of participants | Participants and data sources |
| Methods | 6b | Describe matching criteria (if applicable) | Not applicable |
| Methods | 7 | Clearly define all variables | Data processing and definitions |
| Methods | 8 | Describe data sources/measurement methods | Participants and data sources |
| Methods | 9 | Describe efforts to address bias | Data processing and definitions |
| Methods | 10 | Explain study size determination | Statistical analysis |
| Methods | 11 | Explain handling of quantitative variables | Data processing and definitions |
| Methods | 12a | Describe statistical methods | Statistical analysis |
| Methods | 12b | Methods for subgroup/interactions | Statistical analysis |
| Methods | 12c | Address missing data | Statistical analysis |
| Methods | 12d | Explain sensitivity analyses | Supplementary Material |
| Results | 13a | Report numbers at each stage of study | Results; Supplementary Figure S1 |
| Results | 13b | Give reasons for non-participation | Participants and data sources |
| Results | 13c | Consider use of flow diagram | Supplementary Figure S1 |
| Results | 14a | Describe characteristics of participants | Table 1 |
| Results | 14b | Indicate missing data | Statistical analysis |
| Results | 15 | Report outcome data | Results |
| Results | 16a | Give main results with estimates and precision | Results |
| Results | 16b | Report category boundaries | Methods |
| Results | 16c | Translate relative risk into absolute risk (if relevant) | Not applicable |
| Results | 17 | Report other analyses | Supplementary Material |
| Discussion | 18 | Summarise key results | Discussion |
| Discussion | 19 | Discuss limitations and bias | Limitations |
| Discussion | 20 | Provide interpretation | Discussion |
| Discussion | 21 | Discuss generalisability | Limitations |
| Other information | 22 | Funding | Funding statement |
