# Supplementary material for: High-resolution monitoring reveals fragmented 24-hour light exposure in intensive care units
Source: Crit Care. 2026 Jul 2;30:485. doi: 10.1186/s13054-026-06166-8 (PMC13595780; doi:10.1186/s13054-026-06166-8)
Supplement: Supplementary file 2 [file 13054_2026_6166_MOESM2_ESM.docx]

# Supplementary Materials

List of Supplementary Figures

[Supplementary Figure S1 Flow diagram of patient inclusion	2](#_Toc231332005)

[Supplementary Figure S2 Heatmap of median illuminance day of ICU stay	3](#_Toc231332006)

[Supplementary Figure S3 Sensitivity of event rates to threshold choice	4](#_Toc231332007)

[Supplementary Figure S4 Seasonal variation in ICU ambient light exposure	6](#_Toc231332008)

[Supplementary Figure S5 Ambient light exposure by IMV status	7](#_Toc231332009)

[Supplementary Figure S6 Ambient light exposure by NIV status	8](#_Toc231332010)

[Supplementary Figure S7 Ambient light exposure by RRT status	9](#_Toc231332011)

[Supplementary Figure S8 Nocturnal light fragmentation across sedation categories	10](#_Toc231332012)

List of Supplementary Tables

[Supplementary Table S1 Severity of illness and organ support by room configuration	5](#_Toc228541914)

Supplementary Figure S1 Flow diagram of patient inclusion

Supplementary Figure S2 Heatmap of median illuminance day of ICU stay

Heatmap showing hourly median illuminance as a function of hour of day (07:00-06:59) and day of ICU stay. Color intensity represents median illuminance on a pseudo-logarithmic scale (log1p). This figure illustrates temporal patterns and potential changes in light exposure over the course of ICU stay (Supplementary Figure S2).

Supplementary Figure S3 Sensitivity of event rates to threshold choice

Distribution of light event rates (events per hour) calculated using three different illuminance thresholds (10, 20, and 50 lux), stratified by room type and by daytime versus night-time. Event rates are displayed on a pseudo-logarithmic scale (log1p), demonstrating the robustness of fragmentation patterns across threshold definitions

| Severity of illness and organ support by room configuration | | | | | |
| --- | --- | --- | --- | --- | --- |
|  |  |  | Organ support (patient-day level) | | |
|  | patients, n | SAPS III, median (IQR) | invasive mechanical ventilation, % | non-invasive mechanical ventilation, % | renal replacement therapy, % |
| Double room (double occupancy) | 131 | 50.4 (23.9-68.7) | 48.5 | 5.4 | 10.0 |
| Double room (single occupancy) | 44 | 44 (23.9-66.6) | 53.3 | 2.1 | 13.8 |
| Single room | 47 | 52.4 (30.1-71.8) | 35.2 | 3.8 | 16.5 |

Supplementary Table S1 Severity of illness and organ support by room configuration

Severity of illness, measured by SAPS III, did not differ significantly between room types. In contrast, the use of invasive ventilation and renal replacement therapy varied across room configurations, whereas non-invasive ventilation rates were low in all groups

Supplementary Figure S4 Seasonal variation in ICU ambient light exposure

Distribution of ambient illuminance stratified by season. Violin plots with embedded boxplots show median values and interquartile ranges on a logarithmic scale. Median illuminance was lowest in winter and highest in summer, with statistically significant differences between seasons (Kruskal–Wallis test, p < 0.001).

Supplementary Figure S5 Ambient light exposure by IMV status

Distribution of median illuminance levels during daytime (07:00–20:59) and nighttime (21:00–06:59) according to invasive mechanical ventilation (IMV) status. Violin plots represent the density distribution of patient-days and patient-nights, respectively. Boxplots indicate medians and interquartile ranges (IQRs). Illuminance is displayed on a logarithmic scale.

Supplementary Figure S6 Ambient light exposure by NIV status

Distribution of median illuminance levels during daytime (07:00–20:59) and nighttime (21:00–06:59) according to non-invasive ventilation (NIV) status. Violin plots represent the density distribution of patient-days and patient-nights, respectively. Boxplots indicate medians and interquartile ranges (IQRs). Illuminance is displayed on a logarithmic scale.

Supplementary Figure S7 Ambient light exposure by RRT status

Distribution of median illuminance levels during daytime (07:00–20:59) and nighttime (21:00–06:59) according to renal replacement therapy (RRT) status. Violin plots represent the density distribution of patient-days and patient-nights, respectively. Boxplots indicate medians and interquartile ranges (IQRs). Illuminance is displayed on a logarithmic scale.

Supplementary Figure S 8 Nocturnal light fragmentation across sedation categories

Sedation depth was categorized according to mean daily Richmond Agitation-Sedation Scale (RASS) scores as deep sedation (RASS ≤ −3), light sedation/awake (RASS > −3 to ≤ 0), and agitated (RASS > 0). Boxes indicate interquartile ranges and centre lines indicate medians; points represent individual patient-nights. No significant differences in the number of nocturnal light events exceeding 20 lx were observed across sedation categories (Kruskal–Wallis χ² = 1.17, p = 0.556).
